# Whole-genome sequencing of SARS-CoV-2 from residual viral RNA present on positive rapid antigen test kits for genomic surveillance

**DOI:** 10.5365/wpsar.2025.16.1.1140

**Published:** 2025-03-31

**Authors:** Mohd Ishtiaq Anasir, Khayri Kamel, Nor Malizza G Adypatti, Mohammad Syafiq Jamaluddin, Farah Amira Ahmad, Siti Nurhidayah Norhisham, Muhammad Zulfazli Mohamad Sukri, Nur Rafiqah Rosli, Siti Norazrina Saif, Nurul Izzati Basarudin, Mohamad Azzam-Sayuti, Akmal Hayat Abdul Karim, Mahirah Kamil Puat, Ravindran Thayan, Rozainanee Mohd Zain

**Affiliations:** a Virology Unit, Infectious Diseases Research Centre, Institute for Medical Research, National Institutes of Health, Ministry of Health, Selangor, Malaysia.; b Pathology Department, Hospital Tengku Ampuan Rahimah, Selangor, Malaysia.

COVID-19 is a highly infectious disease caused by severe acute respiratory syndrome coronavirus 2 (SARS-CoV-2). (*1*) Since the onset of the COVID-19 pandemic in 2020, SARS-CoV-2 genomic surveillance has been implemented to guide public health responses, initiate early detection and characterization of emerging variants, and understand the impact of emerging mutations on vaccine efficacy. Multiple SARS-CoV-2 variants such as the variants of concern Alpha, Beta, Gamma, Delta and Omicron have emerged. (*2*, *3*) Surveillance of circulating SARS-CoV-2 variants is performed through whole-genome sequencing (WGS) of residual viral transport media previously tested positive by real-time reverse transcription-polymerase chain reaction (RT–PCR). (*3*) However, since the update of the interim guidance by the World Health Organization in October 2021 regarding the use of rapid antigen test kits (RTK-antigen) for the diagnosis of SARS-CoV-2 infection, (*4*) the majority of COVID-19-positive cases are currently diagnosed by this method. In Malaysia, the Ministry of Health promoted self-testing using RTK-antigen as the country began transitioning to the endemic phase in April 2022. (*5*) Thus, genomic surveillance has become more challenging, as COVID-19-positive patients may need to undergo PCR retesting solely for the purpose of WGS. Furthermore, opportunities for SARS-CoV-2 genomic surveillance to identify circulating variants are constrained, as WGS laboratories receive fewer residual PCR-positive clinical samples.

RTK-antigen has emerged as the primary diagnostic tool for detecting SARS-CoV-2 infection, especially in low- and middle-income countries. (*6*) To circumvent the challenges related to a reduction in residual PCR-positive clinical samples, several groups have attempted to recover SARS-CoV-2 ribonucleic acid (RNA) from positive RTK-antigen cassettes for WGS. (*6*-*8*) In this study, we adopted an approach to recover SARS-CoV-2 RNA from RTK-antigen cassettes for WGS of SARS-CoV-2.

## Methods

### Extraction of SARS-CoV-2 RNA from positive RTK-antigen cassettes

In this study, we adopted and modified a method from a previous study to extract SARS-CoV-2 RNA from positive RTK-antigen cassettes. (*8*) Thirty-three leftover ProDetect^TM^ COVID-19 Antigen Rapid Test cassettes (Mediven, Penang, Malaysia) that tested positive from 27 July to 11 August 2022 were collected from a hospital in Klang district, Malaysia. COVID-19 diagnostic testing was routinely performed as part of clinical care using RTK-antigen assay and nasopharyngeal swabs at this hospital. The cassettes were sealed separately in biohazard specimen bags and transported on ice within 48 hours to the sequencing laboratory, where they were processed immediately upon receipt.

**Fig. 1:**
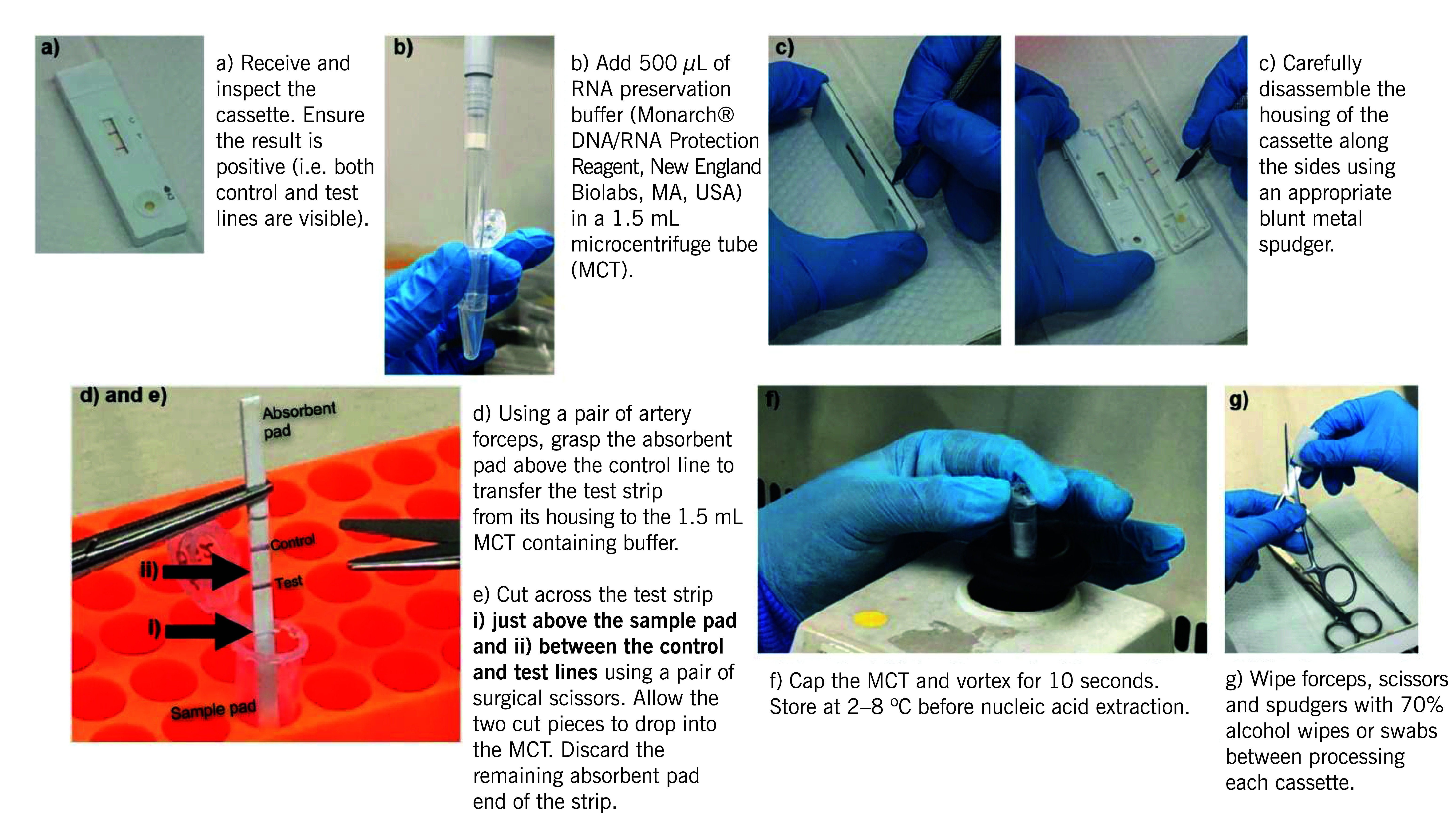
Methodological workflow for RTK-antigen cassette processing

Each cassette was disassembled. The lateral flow strip was transferred to a microcentrifuge tube containing 500 µL of a nucleic acid preservation buffer (Monarch® DNA/RNA Protection Reagent, New England Biolabs, MA, USA), which was diluted to 1:1 from its 2x concentrate (**Fig. 1**).

[**insert** Figure 1]

The microcentrifuge tube, containing both the strip and the nucleic acid preservation buffer, was stored at 2–8 °C before nucleic acid extraction. The period between the collection of samples and nucleic acid extraction in this study was 3–16 days. Before RNA extraction, the strip fragments obtained from the RTK-antigen cassettes were stored in RNA preservation buffer for a maximum of 15 days (**Table 1**).

**Table 1:** Duration of strip storage in sample RNA preservation buffer, Ct value for N gene, RNA quantity after PCR amplification, depth of coverage, genome coverage and Pango lineage of all study samples (*n* = 33)

Sample ID	Duration of strip storage in sample RNA preservation buffer (day)	Ct value for N gene	RNA quantity after amplification (ng/µL)	Genome coverage (%)^a^	Pango lineage
**LF00001**	**1**	**28.09**	**25.0**	**99.04**	**BA.2.38**
**LF00002**	**1**	**27.25**	**28.0**	**99.13**	**BA.5.6**
**LF00003**	**1**	**30.87**	**8.8**	**95.59**	**BA.5.2**
**LF00004**	**1**	**30.4**	**12.0**	**98.42**	**BA.5.2**
**LF00005**	**1**	**35.66**	**3.8**	**53.00**	**N/A**
**LF00006**	**2**	**N/A**	**1.3**	**N/A**	**N/A**
**LF00007**	**2**	**33.38**	**3.1**	**49.58**	**N/A**
**LF00008**	**2**	**31.12**	**3.6**	**99.23**	**BA.5.2.1**
**LF00009**	**2**	**34.52**	**4.9**	**N/A**	**N/A**
**LF00010**	**15**	**30.42**	**0.8**	**18.02**	**N/A**
**LF00011**	**13**	**23.84**	**0.8**	**82.89**	**BA.5.2**
**LF00012**	**12**	**30.48**	**0.8**	**88.84**	**BA.5.2**
**LF00013**	**12**	**30.24**	**0.8**	**83.38**	**BA.5.2**
**LF00014**	**13**	**32.9**	**1.3**	**24.46**	**N/A**
**LF00015**	**12**	**23.47**	**1.0**	**99.19**	**BA.5.2**
**LF00016**	**12**	**32.87**	**0.8**	**3.47**	**N/A**
**LF00017**	**9**	**27.96**	**2.2**	**99.25**	**BA.5.2**
**LF00018**	**9**	**24.6**	**3.3**	**99.03**	**BA.5.2**
**LF00019**	**10**	**28.91**	**2.7**	**99.04**	**BA.5.2.1**
**LF00020**	**10**	**31.9**	**0.8**	**7.18**	**N/A**
**LF00021**	**9**	**25.75**	**3.4**	**99.05**	**BA.5.2**
**LF00022**	**9**	**30.17**	**0.8**	**95.93**	**BA.5.2**
**LF00023**	**9**	**37.62**	**0.8**	**24.43**	**N/A**
**LF00024**	**6**	**32.47**	**0.8**	**39.33**	**N/A**
**LF00025**	**5**	**N/A**	**0.8**	**N/A**	**N/A**
**LF00026**	**5**	**N/A**	**0.8**	**N/A**	**N/A**
**LF00027**	**5**	**37**	**0.8**	**7.31**	**N/A**
**LF00028**	**5**	**30.71**	**0.8**	**82.37**	**BA.5.2**
**LF00029**	**4**	**31.72**	**0.8**	**3.62**	**N/A**
**LF00030**	**1**	**29.21**	**0.8**	**89.70**	**BA.5.2**
**LF00031**	**2**	**25.99**	**2.0**	**95.59**	**BA.5.3**
**LF00032**	**1**	**35.1**	**0.8**	**7.02**	**N/A**
**LF00033**	**2**	**25.24**	**1.0**	**95.93**	**BA.5.2**
**NC**	**N/A**	**N/A**	**0.9**	**N/A**	**N/A**

Ct: cycle threshold; ID: identification; N/A: not available; RNA: ribonucleic acid.

^a^ Variant filtering during the analysis with wf-artic Nextflow mandates a minimum coverage of at least 20x at variant/genotyping loci for a call to be made.

MagMAX Viral/Pathogen Nucleic Acid Isolation Kit (Thermo Fisher Scientific, MA, USA) was used to extract 400 µL of the nucleic acid preservation buffer contained within the microcentrifuge tube. Nucleic acid extraction was performed according to the manufacturer's instructions on the KingFisher Apex (Thermo Fisher Scientific) automated sample purification system, with a final elution volume of 60 µL.

### Detection of SARS-CoV-2 by real-time RT–PCR

RT–PCR was performed using a real-time fluorescent RT–PCR kit for detecting SARS-CoV-2 (MFG030015; BGI Europe A/S, Copenhagen, Denmark), targeting the ORF1ab and N genes of SARS-CoV-2. Ten microlitres of the extracted nucleic acid were added to the RT–PCR master mix, and CFX96 Touch Real-Time PCR Detection System (Bio-Rad Laboratories Inc., CA, USA) was used for thermal cycling as follows: 50 °C for 20 minutes and 95 °C for 5 minutes, followed by 45 PCR cycles of 95 °C for 15 seconds and 60 °C for 30 seconds. Cycle threshold (Ct) values detected for the N gene were recorded for subsequent analysis.

### SARS-CoV-2 genomic sequencing and bioinformatic analysis

Reverse transcription, amplification and library preparation of the SARS-CoV-2 genome for sequencing were performed using Oxford Nanopore Technologies (Oxford, United Kingdom of Great Britain and Northern Ireland) kits inclusive of the Midnight Expansion Kit with Midnight-ONT/V3 primers (EXP-MRT001.30) and Rapid Barcoding Kit 96 (SQK-RBK110.96), as per the protocol outlined by Oxford Nanopore Technologies. (*9*) After quantification of the library using the Invitrogen Qubit 1X dsDNA BR Assay Kit (Q33265, Thermo Fisher Scientific), a total of 800 ng of the library was incubated for 5 minutes at room temperature with 1 µL of Rapid Adaptor F and loaded onto an R9.4.1 flow cell (FLO-MIN106D) for sequencing on an Oxford Nanopore Technologies GridION (MinKNOW v22.05.7) for 72 hours with live high-accuracy (minimum q-score of 9) model basecalling (Guppy v6.1.5). Upon completion of sequencing and basecalling, the FASTQ data were automatically analysed to generate a SARS-CoV-2 consensus sequence using wf-artic Nextflow (v.0.3.14), an automated bioinformatic analysis pipeline provided by Oxford Nanopore Technologies. Variant calling was made with a minimum of 20x coverage, and lineage assignment was made using software version pangolin v4.1.1. Genome coverage or completeness was calculated using nextclade v2.5.0 by identifying the number of Ns in relation to the SARS-CoV-2 reference sequence (NCBI Reference Sequence: NC_045512.2). Consensus sequences with genome coverage of 70% and above were uploaded to the GISAID EpiCoV database.

## Results

### Detection of SARS-CoV-2 RNA extracted from positive RTK-antigen cassettes

The RNA recovered from 33 RTK-antigen cassettes that showed positive results for SARS-CoV-2 was evaluated using real-time RT–PCR to determine the Ct values of the samples. Of the 33 samples, 30 (90.9%) were positive for SARS-CoV-2 by RT–PCR. The Ct values from these RNAs ranged between 23.47 and 37.62 for the N gene (**Table 1**). Three of the samples (LF0006, LF00025, LF00026) did not yield any Ct values.

A Spearman's rank correlation coefficient (*r*^s^) analysis revealed a statistically non-significant and very weak negative correlation between Ct values obtained from real-time RT–PCR and the duration of strip storage in sample RNA preservation buffer (*r^s^* = −0.1349; *P* > 0.05). The three samples that did not yield Ct values were excluded from Spearman’s rank correlation coefficient analysis.

### Whole-genome sequencing of RNA recovered from positive RTK-antigen cassettes

Eighteen positive samples (60%) were successfully sequenced to a reasonable genome coverage: 13 achieved more than 90% genome coverage, and five achieved 80–90% genome coverage, with depth of coverage of at least 20x (**Table 1**). Lineage assignment was successful for all 18 samples. Quantification of RNA from these samples after PCR amplification during library preparation showed that 10 of the 18 samples had RNA quantities in the range of 2.0–28.0 ng/µL, while the other eight samples exhibited RNA quantities similar to the negative control (~0.9 ng/µL). WGS revealed BA.5.2 to be the dominant Omicron subvariant circulating in Malaysia during the study period, with 13 of the samples assigned to this subvariant. Other detected subvariants included BA.2.38 (*n* = 1), BA.5.6 (*n* = 1), BA.5.2.1 (*n* = 2) and BA.5.3 (*n* = 1).

**Fig. 2:**
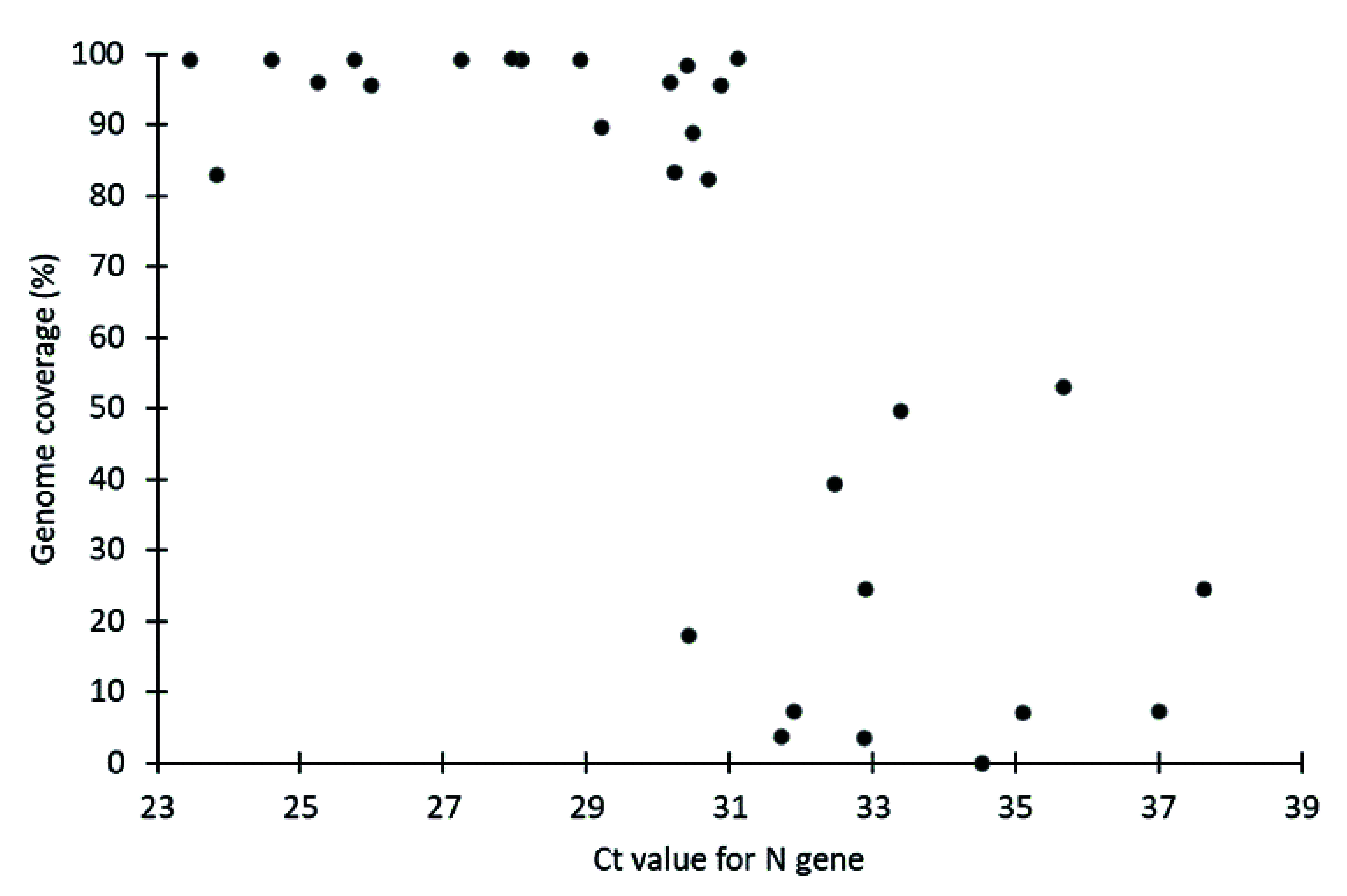
Association between Ct values of SARS-CoV-2-positive samples and genome coverage by whole-genome sequencing (n = 30)

All samples with Ct values in the 20–30 range yielded genome coverage of more than 80% (**Table 1**). The genome coverage significantly diminished for the samples with Ct values of more than 31, with only one of 11 samples in this category achieving genome coverage of more than 80% (**Fig. 2**). Inspection of the quality control parameters for the samples with failed lineage assignment showed that they have a low quantity of starting RNA. Of the 15 samples that failed lineage assignment,  12 exhibited very low RNA quantities ranging from 0.8 to 1.3 ng/µL.

[**insert** Figure 2]

## Discussion

With the advent of genome sequencing technologies, the global genomic surveillance of SARS-CoV-2 was performed in a near real-time fashion. (*10*) For countries that have adopted RTK-antigen as the primary diagnostic tool for COVID-19, we demonstrated that it is feasible to perform genomic surveillance using RNA extracted from SARS-CoV-2 RTK-antigen cassettes. An adequate quantity and reasonable quality of RNA, suitable for targeted sequencing using Oxford Nanopore Technologies, can be obtained from these cassettes.

Ideally, samples should be extracted and sequenced quickly. However, sequencing is typically done in batches to reduce costs, with around 48 to 96 samples per run. This means strip fragments may be stored in RNA preservation buffer for an extended period before extraction. While our RT–PCR data did not show a correlation between storage time and Ct values, further assessment is needed to understand any potential decline in sample quality over time in the buffer.

The sample extraction buffer for RTK-antigen typically includes a phosphate-buffered saline solution with blocking agents, surfactant, lysis agent and preservative. (*11*) The inability to detect SARS-CoV-2 RNA in three samples could be attributed to the absence of an RNase inhibitor in the RTK-antigen buffer. This absence makes the RNA vulnerable to degradation when exposed to the extraction buffer.

Our data showed that WGS can be performed using RNA extracted from RTK-antigen collected as a part of clinical practice, with real-world storage and transport conditions for tropical countries like Malaysia. Our WGS results correlated with the circulating variant during the period of sample collection in Malaysia. Crucially, this study builds on previous proof-of-principle studies and supports the inclusion of RTK-antigen in genomic surveillance. (*6*-*8*)

In resource-limited settings, thoughtful sample selection is critical to ensure a high success rate of SARS-CoV-2 WGS. Thus, evaluating the quality of samples using RT–PCR or Qubit is crucial to avoid wasting resources. Our study suggests that Ct values obtained from RT–PCR can be a good indicator for predicting the success of WGS. Samples with Ct values of < 31 are optimal for inclusion in WGS. The limitations of our study include the small sample size, consisting of only 33 samples, and the short period of sample collection (from 27 July to 11 August 2022). Conducting studies with larger sample sizes and over a longer collection period would enable a more comprehensive evaluation of the feasibility of integrating RTK-antigen cassettes into the genomic surveillance of SARS-CoV-2. Another limitation is that our study used RTK-antigen cassettes from a single commercial brand. While we acknowledge the inability to test all available commercial RTK-antigen cassettes, future experiments should include various other brands to ensure their suitability for inclusion in genomic surveillance programmes. Furthermore, Spearman's rank correlation coefficient analysis revealed that the correlation between Ct values and duration of strip storage in RNA preservation buffer was not statistically significant (*P* > 0.05), indicating that the observed relationship may be due to random variation. Further studies with larger sample sizes are needed to validate these preliminary findings.
